# Atherosclerosis in patients with cervical artery dissection

**DOI:** 10.1177/23969873241274547

**Published:** 2024-09-04

**Authors:** Eveline Brunner, Josefin E Kaufmann, Sandro Fischer, Henrik Gensicke, Annaelle Zietz, Alexandros A Polymeris, Valerian L Altersberger, Philippe A Lyrer, Christopher Traenka, Stefan T Engelter

**Affiliations:** 1 Department of Neurology and Stroke Center, University Hospital Basel and University of Basel, Basel, Switzerland; 2 Neurology and Neurorehabilitation, University Department of Geriatric Medicine FELIX PLATTER, University of Basel, Basel, Switzerland; 3 Department of Clinical Research, University of Basel, Basel, Switzerland; 4 Department of Medicine and Neurology, Melbourne Brain Centre at the Royal Melbourne Hospital, University of Melbourne, Parkville, VIC, Australia

**Keywords:** Atherosclerosis, cervical artery dissection, recurrence, vascular events

## Abstract

**Introduction:**

Cervical artery dissection (CeAD) is considered a non-atherosclerotic arteriopathy, but atherosclerosis of the cervical arteries may co-exist. We explored the frequency and clinical importance of co-existent atherosclerosis in patients with CeAD.

**Patients and methods:**

Single-center exploratory study from the Stroke Center Basel, Switzerland. We re-reviewed duplex ultrasound images at (i) baseline and (ii) last follow-up visit for the presence versus absence of the following atherosclerotic manifestations in the carotid arteries: (i) abnormal carotid intima-media thickness, (ii) plaques, and (iii) atherosclerotic stenosis. We investigated whether CeAD patients with versus without co-existing atherosclerosis differ regarding (a) recurrence of CeAD and (b) occurrence of vascular events (myocardial infarction, peripheral artery disease, or ischemic stroke) using logistic regression with adjustment for age and follow-up time.

**Results:**

Among 294 CeAD patients (median age 46 [IQR 37–53], 41.8% women), 35 (12%) had any atherosclerotic signs at baseline. Among 196 patients with available follow-up, another 21/196 (11%) patients developed atherosclerosis during a median follow-up of 55.7 months. Patients with atherosclerosis had decreased odds of recurrent CeADs when compared to patients without atherosclerosis (OR 0.03, 95% CI = 0.00-0.30). During follow-up, 6 (15%) vascular events occurred among 40 CeAD patients with atherosclerosis and 13 (8.5%) among 153 patients without atherosclerosis (OR 1.38, 95% CI = 0.39-4.55, data for 3 patients were missing).

**Discussion and conclusion:**

Signs of atherosclerosis in the carotid artery were detectable in 12% of CeAD patient at baseline. Additionally, 11% of CeAD patients developed new signs of atherosclerosis within the following 5 years. The presence of atherosclerosis may suggest a lower risk for recurrent CeAD. Whether it might indicate an increased risk for late clinical vascular events deserves further studies.

## Graphical abstract

**Graphical abstract:**
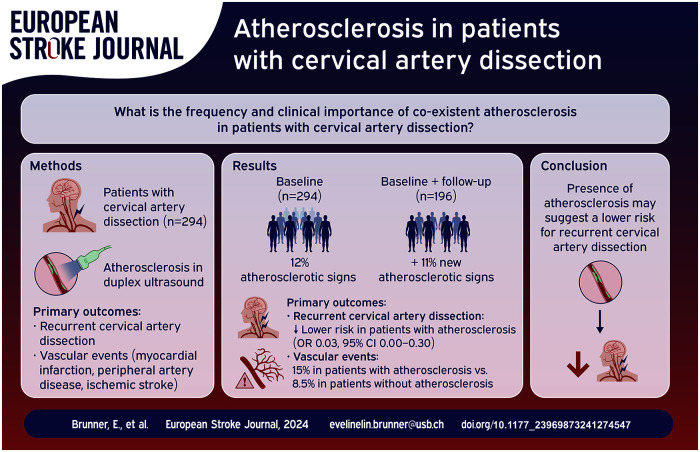

## Introduction

Cervical artery dissection (CeAD) is a leading cause of stroke in the young.^1–3^ CeAD is considered a non-atherosclerotic arteriopathy^4^ with an intramural hematoma as pathophysiologic hallmark, which is thought to be caused by a subintimal tear into the arterial wall of the carotid or the vertebral artery or by rupture of the vasa vasorum.^5^ However, atherosclerosis of the cervical arteries may co-exist^6^ in CeAD. This assumption is supported by the fact, that atherosclerosis^6^ and CeAD^7,8^ share arterial hypertension as major risk factor. Nevertheless, little is known about the frequency and the clinical significance of atherosclerostic manifestations in the cervical arteries among patients with CeAD.

Duplex ultrasound is an established, non-invasive, and widely available diagnostic tool to monitor CeAD patients^3,9^ and also to detect atherosclerosis of the carotid arteries.^10^

In this study, our objective was to examine the frequency of signs of atherosclerosis in the carotid arteries, as identified by duplex ultrasound images, among patients with CeAD at baseline and over time. Particularly, we aimed to determine whether CeAD patients with co-existing atherosclerosis differ from those without atherosclerosis regarding the risk of recurrent CeAD and vascular events of atherosclerotic or embolic origin during follow-up.

## Methods

Study design and patients: This single-center exploratory study is based on data from the CeAD registry of the Stroke Center at the University Hospital Basel, Switzerland, which included all consecutive CeAD patients treated at this center since 1999, as described in prior research.^9^ The diagnosis of CeAD was based upon established and widely accepted diagnostic CeAD criteria,^8,11,12^ including the following arterial findings (at least one): presence of a mural hematoma, aneurysmal dilation, long tapering stenosis, intimal flap, double lumen, or occlusion situated >2 cm above the carotid bifurcation, revealing an aneurysmal dilation or a long tapering stenosis after recanalization.^5,8,9^ For the current analysis, we included all patients enrolled in our CeAD registry until February 2024 who had a baseline duplex ultrasound examination within 1 month after the first symptom attributable to the index CeAD. Based on these baseline duplex ultrasound examinations, we assessed the frequency of signs of atherosclerosis at baseline. To examine the development of atherosclerosis over time, we restricted the study population to those CeAD patients with a long-term follow-up. Therefore, we analyzed the latest follow-up duplex ultrasound examination, which had to be at least 12 months after the baseline duplex ultrasound.

Aims: The current study had the following aims: in CeAD patients (i) to explore the frequency and characteristics of co-existing atherosclerosis in the carotid arteries at baseline and during follow-up, and compare the characteristics of those patients with versus without co-existing atherosclerosis; (ii) to explore whether CeAD patients with co-existing atherosclerosis differ from those without atherosclerosis regarding (a) recurrent CeAD and (b) occurrence of myocardial infarction, peripheral artery disease, or ischemic stroke as vascular events of presumably atherosclerotic or embolic origin.

Review of duplex ultrasound analysis: Duplex ultrasound was conducted in an inpatient or outpatient setting by board-certified stroke neurologists with approved specialization in neurovascular ultrasound. Extracranial and intracranial artery duplex ultrasound was performed with color duplex scanners used in clinical routine (ATL HD5000, Zonare ZS-3, Acuson Sequoia 512, Acuson 128 XP 10C, Philips iU22, Philips EPIQ 5G, Philips Epiq 7), applying linear array (8–3 MHz, 12–5 MHz) probes for extracranial duplex and sector (1–5 MHz) probes for transtemporal and transforaminal duplex ultrasound. All duplex ultrasound examinations were stored in the stroke center’s database and extracted for the present study. Duplex ultrasound images were re-reviewed and evaluated by the following three raters: two independent certified (neurovascular ultrasound) reviewers (C.T. and S.T.E.) and one MD candidate trained in the duplex ultrasound assessment of CeAD and atherosclerosis morphology (E.B.). The raters were not blinded to previous duplex ultrasound results and patient information.

We looked at signs of atherosclerosis in the carotid arteries only, as the assessment of atherosclerosis in the vertebral arteries is associated with great uncertainty. All reviewers assessed the presence or absence of the following ultrasound findings (at baseline and follow-up examination): (i) carotid intima-media thickness (CIMT), (ii) plaque, and (iii) atherosclerotic stenosis. CIMT is defined as the distance from the lumen-intima interface to the media-adventitia interface.^13^ As suggested for standard examination, we analyzed the CIMT of the common carotid artery (CCA) in long-axis view to describe beginning atherosclerosis.^14,15^ A CIMT > 0.9 mm was considered abnormal.^16,17^ A plaque was defined as (i) focal thickening thought to be atherosclerotic in origin protruding into the lumen or (ii) diffuse increase of CIMT in any segment of the carotid artery exceeding the thickness of the surrounding area by >50%.^15^ Identifying the difference between medial thickening and diffuse atherosclerotic plaque is recognized as a matter of debate. Consequently, we followed the recommendations provided by the American Society of Echocardiography.^15^ Each plaque was assessed for echogenicity, and a distinction was made between predominantly hypoechoic or hyperechoic features. Plaques that could not be assigned to either group were termed mixed plaques. Artery stenosis through atherosclerosis was further analyzed with Doppler spectral waveforms and flow parameters to determine the hemodynamic status of the artery. Atherosclerotic stenosis of the carotid artery was classified according to the following criteria as done in prior research^18^: normal (0%–49% stenosis), moderate (50%–69%), or severe (70%–99%). If atherosclerosis was detected, we assessed whether it was located in the artery affected by CeAD.

We also reassessed the arterial characteristics of the dissected arteries on duplex ultrasound at baseline, applying criteria from prior research: (i) mural hematoma,^19,20^ (ii) intimal flap,^20^ (iii) double lumen,^20,21^ (iv) dissecting aneurysm,^20^ (v) stenosis of the dissected artery,^22^ and (vi) occlusion.^23^

Study variables and patient clinical characteristics: Clinical data extracted from the local CeAD database included (1) demographic data (age, sex); (2) site of dissection (internal carotid artery or vertebral artery); (3) presence and type of cerebral ischemic events at baseline (i.e. transient ischemic attack, amaurosis fugax, retinal infarction, or ischemic stroke) and stroke severity as measured by the NIH Stroke Scale (NIHSS; for patients with CeAD who had no cerebral ischemic event, NIHSS of 0 was defined); (4) vascular risk factors (i.e. hypertension, hypercholesterolemia, diabetes, smoking status); (5) treatment of risk factors (i.e. antihypertensives, statins, antithrombotic therapy); (6) vascular events (i.e. myocardial infarction, ischemic stroke, peripheral artery disease (PAD)); (7) and information on the presence or absence of preceding (within 4 weeks prior to the CeAD) severe trauma (i.e. trauma leading to medical examination or hospitalization). The definitions of these variables were previously outlined.^24–28^ The definition of certain risk factors (e.g. hypercholesterolemia) was subject to change during the course of follow-up. Thus, the presence or absence of risk factors was readjusted to the most recent state of guidelines: hypertension (systolic blood pressure > 140 mmHg, diastolic blood pressure > 90 mmHg),^29^ diabetes mellitus (fasting plasma glucose > 7.0 mmol/l (126 mg/dl),^30,31^ hypercholesterolemia (LDL cholesterol (>2.6 mmol/l (>100 mg/dl).^32^

Statistical methods: We compared baseline characteristics of patients with and without signs of atherosclerosis, and descriptively compared the follow-up characteristics. We defined atherosclerosis as beginning atherosclerosis (i.e. increased carotid intima-media thickness) and manifest atherosclerosis (i.e. plaque or atherosclerotic stenosis).

We then assessed the primary outcomes (i) recurrent CeAD (i.e. detection of at least one of the aforementioned CeAD imaging criteria on follow-up duplex ultrasound in a previously unaffected artery based on both the baseline (or prior) duplex ultrasound and the report of the baseline (or prior) MR imaging) and (ii) clinical events of presumably atherosclerotic or embolic cause (i.e. myocardial infarction, peripheral artery disease, ischemic stroke). To effectively handle the low number of primary outcomes, exploratory logistic regression analyses using maximum penalized likelihood method were conducted to investigate the relationship between atherosclerosis and the primary outcomes. Post hoc, we conducted similar logistic regression analyses to investigate the vascular events in cases where both dissection and atherosclerosis occurred in the same cervical artery. For all logistic models, we adjusted for age and follow-up time.

We specifically assessed manifest atherosclerosis (i.e. plaques or atherosclerotic stenosis) in the primary outcomes with absolute numbers in a separate table. To ensure completeness and demonstrate generalizability, we have included a separate table displaying the baseline characteristics of patients who did not have a follow-up ⩾12 months after the index event (Supplemental Table 2). Additionally, to analyze the risk factors associated with recurrent dissection, we have provided an extra table (Supplemental Table 4).

For all results, we provided relative effects (odds ratio (OR) with 95% confidence intervals (CI)). Because of the exploratory nature of the study, no adjustments for multiple testing were made. All statistical analyses were performed using R Studio version 4.3.2 (2023-10-31 ucrt).

Ethics: This study was approved by the Ethics Committee “Ethikkommission Nordwest- und Zentralschweiz” (EKNZ), and patients’ consent was obtained.

## Results

**Figure 1.**
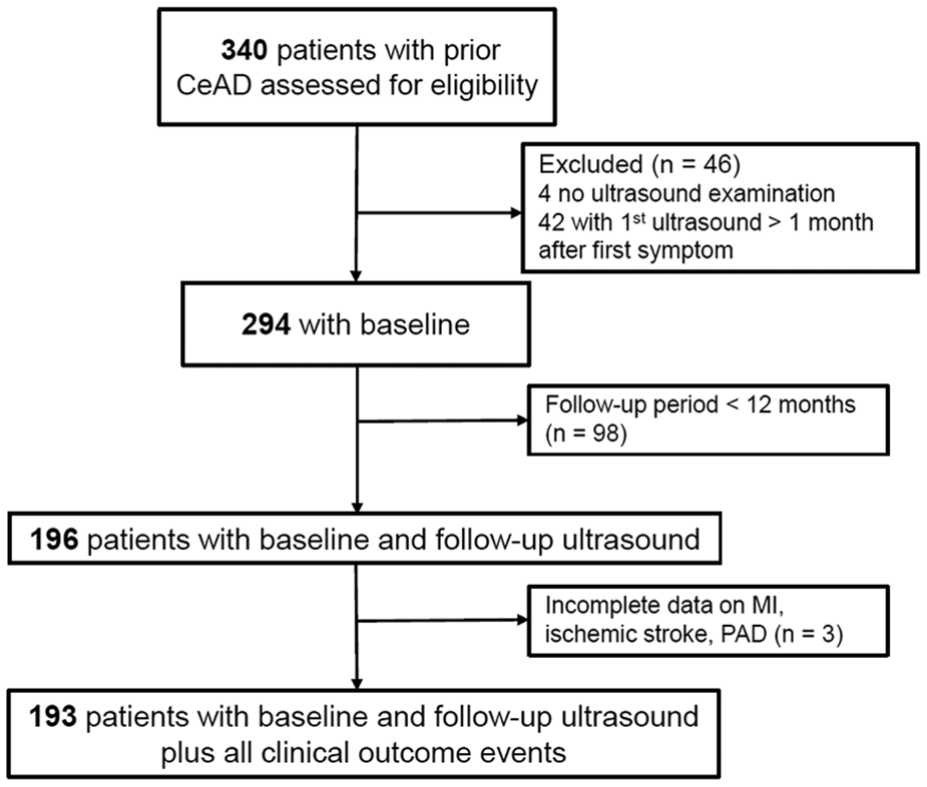
Flowchart of enrolled patients. CeAD: cervical artery dissection; MI: myocardial infarction; PAD: peripheral artery disease.

Patients and baseline characteristics: Among 340 CeAD patients included in the Basel CeAD registry, 294 (86%) met the eligibility criteria and were included in the analyses (Figure 1). Median age of the study population was 46 years (interquartile range (IQR) 37–53) and 41.8% of the population was female. 7/294 (2.4%) patients had a preceding severe trauma. Most patients had a dissection of the internal carotid artery (69.4%), and dissected artery occlusion at baseline appeared in roughly one-third of the study population (31.0%). At baseline, 170/294 (57.8%) of patients presented with signs or symptoms of a cerebral ischemic event.

35/294 (12%) showed signs of atherosclerosis at baseline. All 35 (100%) of them had an abnormal CIMT, 1 (2.9%) presented with atherosclerotic stenosis ⩾50%, and 16 (45.7%) showed plaques. Most plaques showed mixed echogenicity (8/16, 50%) or were classified as hyperechogenic (7/16, 44%). Among 35 patients with atherosclerosis, 13 (37.1%) had atherosclerosis on the artery affected by CeAD.

Patients with co-existing atherosclerosis at baseline were older (median age 56 [IQR 51.50–62]) and more frequently male patients (80% of 35) than patients without atherosclerosis (median age 44 [IQR 37–52], 55.2% male). Vascular risk factors were more prevalent in patients with co-existing atherosclerosis than in those without (hypertension in 51.4% vs 31.0%, hypercholesterolemia in 34.3% vs 12.8%, and smoking at index event in 40.0% vs 27.2%). Diabetes mellitus was present in only a minimal percentage of the study population (3/294 (1%); Table 1).

**Table 1.** Patient baseline characteristics and comparisons among those with atherosclerosis and without (*n* = 294).

Baseline characteristics	Atherosclerosis at baseline	No atherosclerosis at baseline	Missing
Number of patients, *n* (%)	35 (12)	259 (88)	0
Sex, female, *n* (%)	7 (20.0)	116 (44.8)	0
Age at baseline, years (median, IQR)	56 [51.50, 62.00]	44 [37.00, 52.00]	0
Etiology CeAD: severe trauma, *n* (%)	2 (5.7)	5/258 (1.9)	0.4%
Site of dissection			
Internal carotid artery dissection, *n* (%)	25 (71.4)	179 (69.1)	0
Vertebral artery dissection, *n* (%)	10 (28.6)	84 (32.4)	0
Ultrasound characteristic of dissection			
Intima flap, *n* (%)	4 (11.4)	33 (12.7)	0
Double lumen, *n* (%)	0 (0.0)	5 (1.9)	0
Wall hematoma, *n* (%)	7 (20.0)	93 (35.9)	0
Stenosis through dissection, *n* (%)	10 (28.6)	84 (32.4)	0
Occlusion at baseline, *n* (%)	11 (31.4)	80 (30.9)	0
Ultrasound characteristic of atherosclerosis			
Intima-media thickened, *n* (%)	35 (100)	-	0
Atherosclerotic stenosis, *n* (%)	1 (2.9)	-	0
Plaques, *n* (%)	16 (45.7)	-	0
Hypoechogenic plaque, *n* (%)	1 (2.9)	-	0
Hyperechogenic plaques, *n* (%)	7 (20.0)	-	0
Mixed plaques, *n* (%)	8 (22.9)	-	0
Atherosclerosis in CeAD vessel	13 (37.1)	-	0
Cerebral ischemic event at baseline, *n* (%)	22 (62.9)	148 (57.1)	0
NIHSS score at admission, median (median, IQR)	1.00 [0.00, 3.50]	0.00 [0.00, 2.50],*	1.4%
Risk factors			
Hypertension, *n* (%)	18 (51.4)	80/258 (31.0)	0.4%
Hypercholesterolemia, *n* (%)	12 (34.3)	33/258 (12.8)	0.4%
Diabetes mellitus, *n* (%)	2 (5.7)	1/258 (0.4)	0.4%
Smoking at index event, *n* (%)	14 (40.0)	70/257 (27.2)	0.8%
Past smoking, *n* (%)	6 (17.1)	55/257 (21.4)	0.8%
Secondary prevention			
Hypertension with treatment, *n* (%)	18 (51.4)	76/258 (29.5)	0.4%
Statin therapy before event, *n* (%)	6 (17.1)	5/257 (1.9)	0.8%
Antiplatelets, *n* (%)	27 (77.1)	193/256 (75.4)	1.2%
Anticoagulants, *n* (%)	17 (48.6)	93/256 (36.3)	1.2%

IQR: interquartile range; NIHSS: National Institutes of Health Stroke Scale.

* Refers to *n* = 255, data for four patients are missing.

Follow-up characteristics: Of the 294 patients with baseline duplex ultrasound, 196 patients had a long-term follow-up of at least 12 months (Figure 1). Among these, 21 (11%) showed signs of atherosclerosis at baseline, and another 21 (11%) patients developed atherosclerosis in the period between baseline and follow-up, resulting in a total of 42 (21%) atherosclerotic CeAD patients (Supplemental Table 1). Median follow-up time was 55.7 months, ranging from 1 to 22 years, and CeAD patients with co-existing atherosclerosis had a longer follow-up time (57 months [IQR 22.25–119]) compared to patients without atherosclerosis (35 months [IQR 16.25–65.75]). Plaques were observed in 47.6% of atherosclerotic CeAD patients, and most of the plaques were observed to be hyperechogenic (12/20, 60%). Only 2 patients (4.8%) showed atherosclerotic stenosis ⩾50%. CeAD patients with co-existing atherosclerosis more frequently had hypercholesterolemia than patients without atherosclerosis (15.0% vs 10.0%; Table 2).

**Table 2.** Patient characteristics at follow-up and comparisons among those with atherosclerosis and without (*n* = 196).

Characteristics at follow-up	Atherosclerosis	No atherosclerosis	Missing
Number of patients, *n* (%)	42 (21)	154 (79)	0
Age at follow-up, years (median, IQR)	61 [53.00, 69.75]	47 [40.00, 53.00]	0
Follow up time, months (median, IQR)	57 [22.25, 119.00]	35 [16.25, 65.75]	0
Ultrasound characteristics of atherosclerosis			
Occlusion at follow-up	7 (16.7)	23 (14.9)	0
Intima-media thickened, *n* (%)	42 (100.0)	-	0
Atherosclerotic stenosis, *n* (%)	2 (4.8)	-	0
Plaques, *n* (%)	20 (47.6)	-	0
Hypoechogenic plaque, *n* (%)	1 (2.4)	-	0
Hyperechogenic plaques, *n* (%)	12 (28.6)	-	0
Mixed plaques, *n* (%)	9 (21.4)	-	0
Atherosclerosis in CeAD vessel	23 (54.8)	-	0
Newly developed risk factors			
Hypertension	8 (19.0)	29/152 (19.1)	1.0%
Hypercholesterolemia	6/40 (15.0)	15/150 (10.0)	3.0%
Smoking at follow-up	2/40 (5.0)	5/150 (3.3)	3.0%
New secondary prevention			
Hypertension with treatment	10 (23.8)	29/152 (19.1)	1.0%
Statin therapy	18 (42.9)	21/152 (13.8)	1.0%
Past myocardial infarction, stroke or known PAD	6/40 (15.0)	13/153 (8.5)	1.5%
Recurrent cervical artery dissection since index event	0 (0.0)	14 (9.1)	0

IQR: interquartile range; CeAD: cervical artery dissection; PAD: peripheral artery disease.

**Figure 2.**
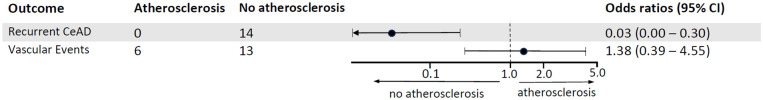
Forest plot: Impact of atherosclerosis on recurrent CeAD and vascular events. CeAD: cervical artery dissection; CI = confidence interval.

Clinical outcomes: 14 recurrent CeADs occurred in the patient group without atherosclerosis (9.1%), and no recurrent CeAD occurred in atherosclerotic CeAD patients (0/42, OR [95% CI] 0.03 [0.00–0.30]). During follow-up, 6/40 (15%, data for 2 patients were missing) vascular events (i.e. myocardial infarction, peripheral artery disease, or ischemic stroke) occurred among atherosclerotic CeAD patients, and 13/153 (8.5%, data for 1 patient was missing) occurred in those without atherosclerosis (OR [95% CI] 1.38 [0.39–4.55]; Table 2, Figure 2). These findings were confirmed in the post-hoc analysis, which investigated the vascular events in cases where both dissection and atherosclerosis occurred in the same cervical artery (OR [95% CI] 2.23 [0.52–8.85]).

Looking at manifest atherosclerosis only, it was detected in 20 of 196 patients with long-term follow-up (10.2%). None (0%) of these patients suffered a recurrent CeAD during follow-up and vascular events occurred in 4/19 (21.1%, data for 1 patient was missing) patients with manifest atherosclerosis (Supplemental Table 3).

## Discussion

This single-center study exploring atherosclerosis in CeAD patients revealed the following key findings: (i) atherosclerosis was present in 12% of CeAD patients at baseline and developed in an additional 11% within the following 4.6 years (55.7 months) after CeAD; (ii) patients with atherosclerosis (either at baseline or during follow-up) had lower odds of recurrent cervical artery dissections; and (iii) myocardial infarction, ischemic stroke, and peripheral artery disease occurred numerically more often in CeAD patients with atherosclerosis than in those without, while this difference did not reach significance.

Our finding that 12% of CeAD patients showed signs of atherosclerosis at baseline was important, as hardly any comparable published data specifically investigating atherosclerosis in CeAD patients are available. Brkić et al. reported a prevalence of atherosclerosis in 17.5% of 188 patients with vertebrobasilar and/or internal carotid artery dissection with a mean age of 53.7 years.^33^ They observed atherosclerosis significantly more often in vertebral artery dissection than in internal carotid artery dissection patients. Chien et al. analyzed atherosclerosis specifically in patients with vertebrobasilar artery dissection in a patient group with a mean age of 65.5 years,^34^ differing from the typical age group of CeAD patients. Age is one of the main risk factors for atherosclerosis^35^ and could thus present a major confounder in the assessment of atherosclerosis in CeAD patients. The median age in our study was 46, which is more representative of a typical CeAD patient population. However, patients with atherosclerosis in our study were also older compared to those without atherosclerosis.

Ibanez et al. reported a prevalence of atherosclerosis detected by ultrasound of the carotid artery in healthy middle-aged subjects (mean age 46 years) of 24% in women and 36% in men.^36^ These higher rates of atherosclerosis in healthy subjects compared to our CeAD patients, the typically young age of occurrence of CeAD,^2^ and the inverse association of CeAD with hypercholesterolemia^37^ all suggest that atherosclerosis is probably not a predisposing condition to CeAD, in contrast with aortic dissection.^38^ Therefore, we hypothesize that CeAD patients are less likely to show signs of atherosclerosis, which is probably supported by the significantly higher occurrence of recurrent CeAD in patients without atherosclerosis. A potential explanation is that as individuals develop atherosclerosis, changes in the structure of the cervical arterial wall enhance its resistance to (intimal) tears. This is also speculated to be due to increased production and decreased breakdown of extracellular matrix components, as well as increasing collagen and elastin cross-links.^37,39,40^ Further, potential confounding factors include the genetic profiles of patients, as genetic studies showed an inverse association between atherosclerosis and CeAD for specific alleles.^41^

Prior studies have shown that atherosclerosis is firmly established as the culprit causing clinical events (i.e. coronary heart disease and stroke).^42,43^ In our study, vascular events of atherosclerotic or embolic origin were more frequent in CeAD patients with co-existing atherosclerosis, although the difference to patients without atherosclerosis was not statistically significant. This is most likely attributed to the limited size of the study population and the small number of vascular events.

Our study has the following limitations: (i) despite performing a thorough review of all data, the analyses are based on retrospectively assessed, non-monitored, non-randomized data, hence raising the risk of chance findings. Furthermore, we had experienced raters, but did not assess the interrater reliability of our results. (ii) The retrospective design of the study with no predefined numbers or time points of follow-up examinations also increases the risk of selection bias, for example, toward patients at risk for complications that may be more likely to be followed up over a longer period of time and meanwhile develop atherosclerosis. (iii) Among other features, we distinguished atherosclerosis through abnormal carotid intima media thickness (CIMT), but as our observations were based on retrospective analyses, exact measurements were faced with technical limitations. (iv) Due to the limited sample size and primary outcomes, our main analyses not only included manifest atherosclerosis but also beginning atherosclerosis. This study should be regarded as an exploratory pilot study, with results that support the need for a larger multicenter study.

The strength of the current research includes the characteristics of our CeAD cohort, which closely resemble those of previous single- as well as multicenter CeAD studies,^22,24^ allowing for generalizability. Furthermore, we used duplex ultrasound as a tool to assess both (i) CeAD characteristics and (ii) atherosclerotic findings at different time points, conducted by raters experienced in both CeAD and atherosclerosis of the carotid arteries.^9^ Due to the relatively young age of the patients at baseline, we were able to include individuals with early-stage atherosclerosis. Lastly, with a relatively long follow-up period, featuring a median of 4.6 years and extending up to 22 years, our study provides longitudinal data, enabling a comprehensive evaluation of the long-term outcomes and progression of CeAD.

In conclusion: Signs of atherosclerosis in the carotid artery were detectable in 12% of CeAD patient at baseline. Additionally, 11% of CeAD patients developed new signs of atherosclerosis within the following 5 years. The presence of atherosclerosis may suggest a lower risk for recurrent CeAD. Whether it might indicate an increased risk for late clinical vascular events deserves further studies. Likewise, individualized antithrombotic and statin therapy in patients with CeAD is to be considered and further explored.

## Supplemental Material

sj-docx-1-eso-10.1177_23969873241274547 – Supplemental material for Atherosclerosis in patients with cervical artery dissection Supplemental material, sj-docx-1-eso-10.1177_23969873241274547 for Atherosclerosis in patients with cervical artery dissection by Eveline Brunner, Josefin E Kaufmann, Sandro Fischer, Henrik Gensicke, Annaelle Zietz, Alexandros A Polymeris, Valerian L Altersberger, Philippe A Lyrer, Christopher Traenka and Stefan T Engelter in European Stroke Journal
