# Supplementary material for: Atherosclerosis in patients with cervical artery dissection
Source: Eur Stroke J. 2024 Sep 4;10(1):198–205. doi: 10.1177/23969873241274547 (PMC11569546; doi:10.1177/23969873241274547)
Supplement: sj-docx-1-eso-10.1177_23969873241274547 – Supplemental material for Atherosclerosis in patients with cervical artery dissection [file sj-docx-1-eso-10.1177_23969873241274547.docx]

**Supplement Table 1** Patient baseline characteristics and comparisons among those with atherosclerosis at baseline, follow-up, and without atherosclerosis (n=196)

|  | Atherosclerosis at baseline | Atherosclerosis new at follow-up | No atherosclerosis |
| --- | --- | --- | --- |
| Number of patients, n(%) | 21 (11) | 21 (11) | 154 (79) |
| Sex, female, n(%) | 4 (19.0) | 8 (38.1) | 79 (51.3) |
| Age at baseline, y (median, IQR) | 59 [53.00, 65.00] | 52 [44.00, 59.00] | 42 [36.25, 49.00] |
| **Site of dissection** | | | |
| Internal carotid artery dissection, n(%) | 14 (66.7) | 18 (85.7) | 107 (69.5) |
| Vertebral artery dissection, n(%) | 7 (33.3) | 3 (14.3) | 48 (31.2) |
| **Ultrasound characteristic of dissection** | | | |
| Intima flap, n(%) | 1 (4.8) | 4 (19.0) | 19 (12.3) |
| Double lumen, n(%) | 0 (0.0) | 2 (9.5) | 3 (1.9) |
| Wall hematoma, n(%) | 2 (9.5) | 6 (28.6) | 57 (37.0) |
| Stenosis through dissection, n(%) | 5 (23.8) | 10 (47.6) | 53 (34.4) |
| Occlusion at baseline, n(%) | 7 (33.3) | 6 (28.6) | 52 (33.8) |
| Cerebral ischemic event at baseline, n(%) | 13 (61.9) | 15 (71.4) | 86 (55.8) |
| NIHSS score at admission, median (median, IQR) | 1.00 [0.00, 3.00] | 1.50 [0.00, 5.50]** | 0.00 [0.00, 2.00]* |
| **Risk factors** | | | |
| Hypertension, n(%) | 11 (52.4) | 9 (42.9) | 39 (25.5)* |
| Hypercholesterolemia, n(%) | 6 (28.6) | 4 (19.0) | 13 (8.5)* |
| Diabetes mellitus, n(%) | 1 (4.8) | 0 (0.0) | 1 (0.7)* |
| Smoking at index event, n(%) | 9 (42.9) | 6 (28.6) | 42 (27.5)* |
| Past smoking, n(%) | 3 (14.3) | 5 (23.8) | 30 (19.6)* |
| **Secondary prevention** | | | |
| Hypertension with treatment, n(%) | 11 (52.4) | 9 (42.9) | 38 (24.8)* |
| Statin therapy before event, n(%) | 3 (14.3) | 1 (4.8) | 2 (1.3)* |
| Antiplatelets, n(%) | 15 (71.4) | 12 (57.1) | 109/151 (72.2) |
| Anticoagulants, n(%) | 11 (52.4) | 12 (57.1) | 61/151 (40.4) |

IQR=interquartile range. NIHSS=National Institutes of Health Stroke Scale.

*refers to n=153, data for one patient is missing

** refers to n=20, data for one patient is missing

**Supplement Table 2** Patient baseline characteristics of patients without follow-up and comparisons among patients with atherosclerosis and without

|  | No Follow-up (n=98) | |
| --- | --- | --- |
|  | Atherosclerosis | No atherosclerosis |
| Number of patients, n(%) | 14 (14) | 84 (86) |
| Sex, female, n(%) | 3 (21.4) | 29 (34.5) |
| Age at baseline, y (median, IQR) | 54.50 [48.50, 56.50] | 45.50 [36.00, 52.25] |
| **Site of dissection** | | |
| Internal carotid artery dissection, n(%) | 11 (78.6) | 54 (64.3) |
| Vertebral artery dissection, n(%) | 3 (21.4) | 33 (39.3) |
| **Ultrasound characteristic of dissection** | | |
| Intima flap, n(%) | 3 (21.4) | 10 (11.9) |
| Double lumen, n(%) | 0 (0.0) | 0 (0.0) |
| Wall hematoma, n(%) | 5 (35.7) | 30 (35.7) |
| Stenosis through dissection, n(%) | 5 (35.7) | 21 (25.0) |
| Occlusion at baseline, n(%) | 4 (28.6) | 22 (26.2) |
| **Ultrasound characteristic of atherosclerosis** | | |
| Intima-media thickened, n(%) | 14 (100) | - |
| Atherosclerotic stenosis, n(%) | 0 (0) | - |
| Plaques, n(%) | 7 (50.0) | - |
| Hypoechogenic plaque, n(%) | 1 (7.1) | - |
| Hyperechogenic plaques, n(%) | 1 (7.1) | - |
| Mixed plaques, n(%) | 5 (35.7) | - |
| Cerebral ischemic event at baseline, n(%) | 9 (64.3) | 47 (56.0) |
| NIHSS score at admission, median (median, IQR) | 0.50 [0.00, 6.00] | 0.00 [0.00, 2.00]* |
| **Risk factors** | | |
| Hypertension, n(%) | 7 (50.0) | 32 (38.1) |
| Hypercholesterolemia, n(%) | 6 (42.9) | 16 (19.0) |
| Diabetes mellitus, n(%) | 1 (7.1) | 0 (0.0) |
| Smoking at index event, n(%) | 5 (35.7) | 22/83 (26.5) |
| Past smoking, n(%) | 3 (21.4) | 20/83 (24.1) |
| **Secondary prevention** | | |
| Hypertension with treatment, n(%) | 7 (50.0) | 29 (34.5) |
| Statin therapy before event, n(%) | 3 (21.4) | 2/83 (2.4) |
| Antiplatelets, n(%) | 12 (85.7) | 72 (85.7) |
| Anticoagulants, n(%) | 6 (42.9) | 20 (23.8) |

IQR=interquartile range. NIHSS=National Institutes of Health Stroke Scale.

*data for 2 patients missing

**Supplement Table 3** Clinical outcomes in patients with manifest atherosclerosis compared to those without (n=196)

|  | Manifest atherosclerosis* | No atherosclerosis | Missing |
| --- | --- | --- | --- |
| Number of patients, n(%) | 20 (10.2) | 176 (89.8) | 0 |
| Past myocardial infarction, stroke or known PAD | 4/19 (21.1) | 15/174 (8.6) | 1.5 |
| Recurrent cervical artery dissection since index event | 0 (0.0) | 14 (8.0) | 0 |

*defined as plaques or atherosclerotic stenosis. PAD=Peripheral Arterial Disease

**Supplement Table 4** Patient characteristics and comparisons among those with recurrent CeAD at follow-up and without (n=196)

|  | Recurrent CeAD | No recurrent CeAD | Missing |
| --- | --- | --- | --- |
| Number of patients, n(%) | 14 (7.1) | 182 (92.9) | 0 |
| **Baseline characteristics** | | | |
| Sex, female, n(%) | 9 (64.3) | 82 (45.1) | 0 |
| Occlusion at baseline, n(%) | 4 (28.6) | 61 (33.5) | 0 |
| Cerebral ischemic event at baseline, n(%) | 8 (57.1) | 106 (58.2) | 0 |
| **Follow-up characteristics** | | | |
| Age at follow-up, y (median, IQR) | 47.00 [44.50, 55.50] | 50.00 [41.00, 56.75] | 0 |
| Follow-up time, m (median, IQR) | 92.50 [74.00, 119.00] | 34.50 [15.00, 65.75] | 0 |
| Hypertension, n(%) | 6 (42.9) | 91/181 (50.3) | 0.5% |
| Hypercholesterolemia, n(%) | 0 (0.0) | 44/176 (25.0) | 3.1% |
| Atherosclerosis at follow-up, n(%) | 0 (0.0) | 42 (23.1) | 0 |
